# Experiences of Assistive Products and Home Care among Older Clients with and without Dementia in Sweden

**DOI:** 10.3390/ijerph191912350

**Published:** 2022-09-28

**Authors:** Johan Borg, Moudud Alam, Anne-Marie Boström, Lena Marmstål Hammar

**Affiliations:** 1 School of Health and Welfare, Dalarna University, SE-791 88 Falun, Sweden; 2 School of Information and Engineering, Dalarna University, SE-791 88 Falun, Sweden; 3 Division of Nursing, Department of Neurobiology, Care Science and Society, Karolinska Institutet, SE-141 83 Huddinge, Sweden; 4 Theme Inflammation and Aging, Karolinska University Hospital, SE-141 86 Stockholm, Sweden; 5 R&D Unit, Stockholms Sjukhem, SE-112 19 Stockholm, Sweden; 6 Division of Caring Sciences, School of Health, Care and Social Welfare, Mälardalen University, SE-721 23 Västerås, Sweden

**Keywords:** assistive products, assistive technology, dementia, home care, home care services, older adults, Sweden

## Abstract

The purpose was to compare selection, use and outcomes of assistive products among older home care clients with and without dementia in Sweden, and to explore the relations between the use of assistive products and perceptions of home care, loneliness and safety. Self-reported data from 89,811 home care clients aged 65 years or more, of whom 8.9% had dementia, were analysed using regression models. Excluding spectacles, 88.2% of them used assistive products. Respondents without dementia were more likely to use at least one assistive product but less likely to use assistive products for remembering. Respondents with dementia participated less in the selection of assistive products, used less assistive products, and benefited less from them. Users of assistive products were more likely to be anxious and bothered by loneliness, to feel unsafe at home with home care, to experience that their opinions and wishes regarding assistance were disregarded by home care personnel, and to be treated worse by home care personnel. The findings raise concerns about whether the needs for assistive products among home care clients with dementia are adequately provided for. They also indicate a need to strengthen a person-centred approach to providing home care to users of assistive products.

## 1. Introduction

Population ageing is a concern across the globe. In the European Union, the number of people aged 65–74 years is projected to increase by 16.6% from 2019 to 2050, while the number of people aged 75–84 years and 85 years or more is projected to expand by 56.1% and 113.9%, respectively [1]. Correspondingly, the number of people with dementia in Europe is expected to rise by 40% from 10 million in 2010 to 14 million in 2030 [2].

‘Ageing in place’ is a global policy, which means that older people regardless of disability shall be able to grow old where they wish to and where they feel safe and in good circumstances [3,4]. In Sweden, this is commonly in their regular homes and with home care service [5]. About 10% of the population aged 65 years or older had a safety alarm in 2021; and nearly 7% of the same population received home care other than safety alarm, food distribution, relief or accompaniment, a proportion that increased to more than 30% among those aged 90 or older [6]. As a result of the ageing in place policy, and because the number of beds at residential settings have decreased in Sweden during the last decades, the proportion of person with dementia has increased in regular housing with home care as support [7,8,9]. The increased number of older persons with home care, and specially the increased number of persons with dementia among them, puts serious demands on the society and the home care organizations to provide high quality care [10]. The WHO Global action plan on the public health response to dementia 2017–2025 states that persons with dementia and their informal carers shall receive the care and support they need to fulfil their potential with dignity, respect, autonomy and equality [4].

High quality care shall be person-centred, and thus a person’s perspective of everyday life and care is of great importance [11,12]. The provision of person-centred care is based on a relationship between the care recipient and the carer, and includes respect for the person’s uniqueness, respect of personal preferences and support for self-determination [11]. This emphasizes the importance of involving persons with dementia in decisions about their care. Persons with dementia commonly express a will to participate in decisions about their care and are often involved early in their disease, but less so in later stages [13]. Additionally, persons with dementia with home care have been found to be less likely to be asked to be involved in their care compared to those without dementia [14].

**Figure 1:**
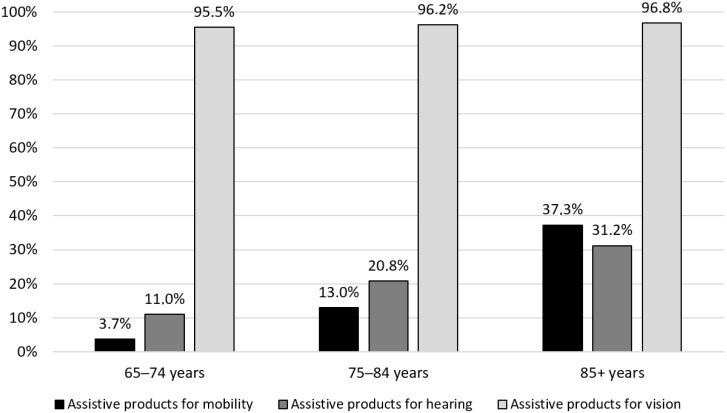
Proportion of older people using assistive products in Sweden by age group [16]. Assistive products for vision include ordinary spectacles.

Besides a growing demand for home care, global initiatives have been taken to improve the access to assistive products to enable older people to continue living at home, delaying or preventing the need for long-term care [15]. Although large proportions of older adults in Sweden use various assistive products (see Figure 1), little is known about the use and benefits of assistive products among home care clients, particularly among those with dementia. Therefore, the objectives of this study were to compare self-reported selection, use and outcomes of assistive products among older home care clients with and without dementia in Sweden, and to explore the relations between the use of assistive products and perceptions of home care, loneliness and safety.

## 2. Materials and Methods

### 2.1. Design Overview

This study used an observational cross-sectional research design.

### 2.2. Setting and Sample

Annually, the Swedish National Board of Health and Welfare (NBHW) invites all clients of home care in ordinary housing in Sweden to participate in a quality evaluation survey. This study used the NBHW survey from 2017 with a target population consisting of 144,643 individuals [17] of which 91,634 responded (response rate 63.4%). The reason for choosing the 2017 survey was that later surveys contained less questions about assistive products. After data cleaning, the researchers were provided with a data set consisting of 89,811 individual responses (i.e., a final response rate of 62.1%). The reason of non-response was largely unknown (91% of the cases) but included unwillingness (5%), sickness, including dementia (2%), and death (0.4%). A large proportion of the questionnaires were completed by proxy respondents (a relative, family member, or home care staff), often together with the primary respondent. After accepting proxy responses, we assumed that the non-response mechanism was completely at random, i.e., we considered the respondents as a random sample from the target population. The validity of this assumption was assessed by comparing sample summary statistics of the respondents’ background characteristics with that of the general population.

### 2.3. Data Collection

NBHW sent a paper questionnaire by post to all home care clients in Sweden aged 65 years or above. The respondents filled in their responses themselves or with the assistance of a proxy. There was also an option to respond via the Internet. The questionnaire contained 24 questions addressing health, contacts with the municipality, home care, loneliness, and assistive products. If a family member acted as a proxy, they were requested to answer a question on cooperation with the home care services.

To identify people with dementia, the NBHW registers on patients and prescribed drugs were used. Individuals who had been diagnosed with dementia were identified through the ICD-10 codes F00-F03 or the medication code N06D in 2017. Data on individuals’ use of safety alarms were retrieved from the NBHW register on care and services for older people and persons with impairments from 2017.

### 2.4. Variables

Table 1 presents the variables, sources of information, questions and response options used in this study.

### 2.5. Statistical Analyses

As the outcome variables of interest were either binary or ordinal, we used logistic regression model for binary response, and proportional odds (cumulative logit) regression model for ordinal response [18] to estimate strengths of association of the outcome variables with the covariates, and in drawing inference on them. We fitted a separate model for each outcome variable. For all the models, the following covariates were used: Dementia status (dummy), Age (in years), Sex (dummy; male and female), self-assessed health (dummy; whether quite poor or very poor), and living alone (dummy). In addition, for the cumulative logit models Safety alarm (dummy, whether a safety alarm was installed in the house), and Use (dummy; whether any assistive technology product was used), were also included as covariates.

All the data manipulation and analyses were carried out using R software (version 4.1.3) [19]. The logistic regression models were fitted by using the *glm* function. The cumulative logit regressions were fitted using *polr* function from *MASS* library [20] in R. The *polr* function uses the linear predictor in the form of *log(Prob(Y_i_ ≤ J)/(1 − Prob(Y_i_ ≤ J))) = α_J_ − X_i_ β* where *Y_i_* is the response for individual *i*, *J* = 1,2,… are the response categories, *α_J_* is an intercept (threshold parameter), *X_i_* is a row vector containing all the covariates (age, sex, etc.), and *β* is a vector of cumulative odds ratio parameter. Due to this specific construction of the linear predictor, a positive *β* parameter implies higher cumulative (adjacent category) odds towards higher response category for increased value of the specific covariate. For further clarification, let us assume that an independent variable $X_{1}$ is increased by one unit, while all other independent variables are kept constant. Then, the proportional odds model implied cumulative odds ratio (COR) is given by

$$COR\left(X_{1}=x+1,X_{1}=x\right)=\frac{\frac{Prob\left(Y_{i}\leq J|X_{1}=x+1,X_{2},\cdots \right)}{1-Prob\left(Y_{i}\leq J|X_{1}=x+1,X_{2},\cdots \right)}}{\frac{Prob\left(Y_{i}\leq J|X_{1}=x,X_{2},\cdots \right)}{1-Prob\left(Y_{i}\leq J|X_{1}=x,X_{2},\cdots \right)}}\;or,\;COR\left(X_{1}=x+1,X_{1}=x\right)=\frac{exp\left(\alpha_{J}-\left(x+1\right)\beta_{1}-X_{2}\beta_{2}-\cdots \right)}{exp\left(\alpha_{J}-x\beta_{1}-X_{2}\beta_{2}-\cdots \right)}\;or,\;COR\left(X_{1}=x+1,X_{1}=x\right)=\exp\left(-\beta_{1}\right)$$

Therefore, the model implies that for a unit change in a covariate the odds of the response to fall in or below any specific response category (*J*) changes with a factor of $\exp\left(-\beta_{1}\right)$. Because of the proportional odds construction, this model also implies that the cumulative odds ratio is the same across all response categories.

## 3. Results

The characteristics of the respondents are given in Table 2. Their mean age was nearly 84 years, two thirds were women and nearly one in eleven had dementia. Compared to the respondents without dementia, a smaller proportion of respondents with dementia used any assistive product, reported good health, lived alone, and had a safety alarm. To a larger extent than respondents without dementia, respondents with dementia used assistive products for remembering. The dementia and non-dementia response groups differ statistically significantly on most of the background characteristics. However, the actual difference between these two groups may be very marginal (except for the use of proxy response, assistive products for mobility, and assistive products for remembering), as seen from the tight confidence intervals of the differences around zero (Table 2).

Odds-ratios regarding use versus no use of assistive products modelled on age, sex, health, dementia and living situation are presented in Table 3. Except for other prescribed assistive products, older respondents were statistically significantly more likely to use all types of prescribed assistive products and safety alarm. Compared to men, women were statistically significantly more likely to use assistive products for seeing and reading, mobility, hygiene and remembering, and safety alarm, and less likely to use assistive products for hearing and communicating. Respondents with poorer health were statistically significantly more likely to use all types of assistive products and safety alarm, especially assistive products for mobility, hygiene and communicating.

Respondents with dementia were statistically significantly less likely to use assistive products for seeing and reading, mobility, hygiene and safety alarms, but they used assistive products for remembering to a much higher degree than respondents without dementia. It can also be noted that respondents living alone were statistically significantly more likely to use assistive products for seeing and reading, and remembering, and to use safety alarm, while they were statistically significantly less likely to use assistive products for mobility and hygiene.

Overall, older age, being a woman, poorer health, living with someone and not having dementia were all statistically significantly associated with having at least one assistive product. Moreover, older age, being a man, poor health, dementia and living alone were statistically significantly associated with less participation in the selection of assistive products.

Results from the fitted cumulative logit models are presented in Table 4. It reveals that respondents who used assistive products were statistically significantly more likely to be anxious, to feel unsafe at home with home care, to be bothered by loneliness, to experience that their opinions and wishes regarding assistance were not considered by personnel, and to be treated worse by personnel. Respondents having a safety alarm installed felt safer living at home with home care to a statistically significant degree.

In analysing whether participation in the selection of assistive products was associated with the extent to which assistive products facilitate daily life, respondents who did not use any assistive product were excluded. Compared to users that did not participate or participated partly in the selection of the assistive products, users that participated fully were statistically significantly more likely to report that their assistive product facilitated their daily life. Moreover, respondents with dementia, being male, of older age, or of poorer health were statistically significantly less likely to report that their assistive products facilitated daily life.

Anxiety and feeling unsafe were statistically significantly associated with dementia, being a woman, younger age, living alone and poorer health. Loneliness was statistically significantly associated with dementia, being a woman, living alone and poorer health. Moreover, respondents with dementia, living alone or poor health experienced to a statistically significant larger degree that their opinions and wishes regarding assistance were not considered by personnel, and that they were not treated well by personnel. Older respondents experienced to a statistically significant degree that the personnel treated them better than what younger respondents did. Except for facilitation, proxy response was found to be a statistically significant factor in that proxy respondents answered more towards dissatisfaction than home care clients themselves.

## 4. Discussion

Using cross-sectional data, this study has compared self-reported selection, use and outcomes of assistive products among older home care clients with and without dementia in Sweden, and explored the relations between use of assistive products and perceptions of home care, loneliness and safety. The findings reveal that home care clients with dementia participated less in the selection of assistive products, used less assistive products, and benefited less from assistive products in daily life. Moreover, users of assistive products—irrespective of dementia status—were more likely to be anxious, to feel unsafe at home with home care, to be bothered by loneliness, to experience that their opinions and wishes regarding assistance were not considered by home care personnel, and to be treated worse by home care personnel.

This study supports previous research that has found that user involvement in the assistive technology provision process is associated with better outcomes [21], and current recommendations for assistive technology provision [22]. Users with dementia participating less in the selection of their assistive products reflects previous findings on home care clients with dementia being less involved in their care [14].

Overall, the use of assistive products among home care clients (9 out of 10) is higher than in the general population in the same age group (1 out of 3), especially with regard to mobility [16,22]. The prevalence of use of assistive products for hearing among home care clients corresponded relatively well to the prevalence in the general population in the same age group (Figure 1), while home care clients used assistive products for mobility to a larger extent than the general population. This is expected as reduced function in mobility is a common reason for home care in the older age group [23]. As the survey among home care clients only include assistive products prescribed by the county or municipality, it does not include most spectacles, which explains the large difference in prevalence of assistive products for seeing and reading compared to assistive products for vision in Figure 1.

Except for assistive products for remembering, home care clients with dementia used less assistive products than those without dementia. This raises concern whether all needs for assistive products among clients with dementia are fully met as their functioning in all functional domains are likely to be the same or worse than those without dementia. The differences between clients with and without dementia were largest for seeing and reading, and mobility. Given that people with dementia are at high risk of falling [24] this may call for further studies as to whether falling can be prevented by meeting the needs for assistive products.

Home care clients with dementia benefiting less from their assistive products than did those without dementia may partly be explained by the fact that they participated less in the selection of the assistive products. However, this also calls for further studies to understand why people with dementia do not benefit equally much from using assistive products. One reason may be that they are not able to express their needs to the same extent as people without dementia [25]. This may have implications not only for the provision process, including user training, but also for the design of assistive products.

Home care clients using assistive products were more likely to be anxious and bothered by loneliness than those not using assistive products. A dementia diagnosis includes a diversity of symptoms and one is anxiety, which justifies our results [26]. It may also be understood that the persons with more needs of assistive products probably have poorer health, more functional and cognitive impairments and thereby are more vulnerable and dependent on help and support from formal and informal caregivers as our findings highlight. A previous study has found that persons with dementia having home care services reported to a greater extent that they were not treated with respect and dignity compared to persons without dementia [14]. Moreover, persons with dementia have expressed the importance of being supported as unique and capable humans despite their needs of support and care, and that they wished for self-determination by being informed about and involved in their care [27].

From a perspective of person-centred care, it is alarming that users of assistive products were more likely to feel unsafe at home with home care, to experience that their opinions and wishes regarding assistance were not considered by home care personnel, and to be treated worse by home care personnel. Home care services in Sweden need to take these findings seriously and improve their services, for example, by ensuring that users of assistive products are treated well, feel safe, and participate in decisions about their care in line with person-centred care [11,12,13].

### Discussion of Methods

A strength of this study is the large sample, representing more than 60% of the population aged 65 years or more receiving home care in Sweden. The descriptive statistics of background variables do not indicate any serious fluctuation from the underlying population. Therefore, the assumption of completely random missing data seems to be reasonable. However, there were remarkable difference in respondent’s profile between dementia and the non-dementia group (see Table 2) which justifies controlling for respondent’s background in further statistical analysis.

NBHW collects data using the concerned questionnaire annually and has reported that the results are robust from year to year, indicating good reliability [28]. Relatively large proportions of the responses were provided in the presence of or with support from a proxy, which could be a family member, friend, legal guardian, or home care personnel. Several studies have explored the correlation between self-reported and poxy-reported data. A systematic review found that relatives and staff proxy ratings of quality of life among care home residents with dementia shared a clear relationship [29], while a more recent study reported that family were more likely than staff to rate the quality of life of care home residents with dementia lower [30]. Another study reported that professional and informal carers rated the quality of life of people with dementia lower than they did themselves [31]. For older adults without dementia, one study found that at group level, caregivers proxy reports of patient-reported outcomes tend to approximate patient reports [32]. These studies lend support to our findings, stressing the importance of controlling for proxy reporting, especially for people with dementia. However, previous studies of the NBHW survey analysing self-reported data only and self- and proxy reported data did not find large differences between the data sets [33,34].

Logistic and cumulative logit regression were chosen because they are the most frequently used regression models for analysing binary and ordinal response, respectively. The main motivation being that these models facilitate easy interpretation of the effects of the outcome variables on the binary (ordinal) response through odds (cumulative odds) ratio explanation of the exponential of the model parameter estimates. Except for the model for Treatment and Facilitation, the other models (Table 3) showed large deviance statistic compared to the sample size (deviance/df > 1.5 while its expected value is 1) indicating poor fit of these models with the data. Model fit may be improved by adding more outcome variables, and possibly by changing model formulation. However, because we are only concerned about drawing inference on certain covariate effect, with limited access to confounding factors, we did not attempt to explore possible ways to improve the model fits. For simplicity only the cumulative odds ratios are reported. Most of the covariates are found to be statistically significant. However, the statistical significance may well be an artifact of large sample size. Therefore, we need to emphasize more on the size of the effect than its statistical significance.

## 5. Conclusions

Older home care clients with dementia in Sweden participated less in the selection of assistive products, used less assistive products, and benefited less from them compared to those without dementia. To a greater extent than home care clients not using assistive products, users were anxious and lonely. They also felt more unsafe with home care, experienced that their opinions and wishes were disregarded by home care personnel, and were treated worse by home care personnel.

The findings raise concerns about whether the needs for assistive products among home care clients with dementia are adequately provided for. They also indicate a need to strengthen a person-centred approach to providing home care to users of assistive products. Further studies that explore met needs and reasons for unmet needs for assistive products among people with dementia are called for, as well as of why users of assistive products had worse experiences of home care than non-users.

## Figures and Tables

**Table 1:** List of variables.

Variable	Question ^1^ (Source)	Response Options
Age	(National Patient Register)	Age in years
Sex	(National Patient Register)	1 = Man, 2 = Woman
Dementia	(National Patient Register: ICD−10 codes F00-F03; medication code N06D)	0 = No dementia, 1 = Dementia
Health	How do you judge your general health status?	1 = Very good, 2 = Quite good, 3 = Fair, 4 = Quite poor, 5 = Very poor
Living situation	Do you live together with another adult?	1 = Yes, 2 = No
Anxiety	Are you bothered by anxiety, worry or anguish?	1 = No, 2 = Yes, slightly, 3 = Yes, severely
Safety	How safe or unsafe do you feel living at home with home care service?	1 = Very safe, 2 = Quite safe, 3 = Neither safe nor unsafe, 4 = Quite unsafe, 5 = Very unsafe
Loneliness	Does it happen that you are bothered by loneliness?	1 = Yes, often, 2 = Yes, now and then, 3 = No
Respect	Do the personnel consider your opinions and wishes regarding how the assistance should be performed?	1 = Yes, always, 2 = Most of the time, 3 = Sometimes, 4 = Seldom, 5 = No, never
Treatment	Do the personnel treat you well?	1 = Yes, always, 2 = Most of the time, 3 = Sometimes, 4 = Seldom, 5 = No, never
Participation	Did you get an opportunity to participate in the selection of the assistive product(s)?	1 = Yes, 2 = Partly, 3 = No
Use	Do you use any assistive product that you have got prescribed by the municipality or county?	1 = Seeing and reading (spectacles is not a prescribed assistive product), 2 = Hearing, 3 = Communicating, 4 = Mobility, 5 = Hygiene, 6 = Remembering, 7 = Other prescribed assistive product, 8 = No
Facilitation	Does/Do your assistive product(s) facilitate your daily life?	1 = Yes, 2 = Partly, 3 = No
Proxy	Who were involved in answering the questionnaire?	1 = Homecare client, 2 = Proxy (relative, friend, personnel, etc.)
Safety alarm	(National Register on Care and Services for the Elderly and Persons with Impairments)	0 = No, 1 = Yes

^1^ Authors’ translation of the original Swedish questions in the NBHW quality evaluation survey.

**Table 2:** Comparative background characteristics of the respondents with and without dementia.

Background Variable	People with Dementia	People without Dementia	Total	CI of Difference (Column 2 vs. 3) *
Number (% of total)	7,988 (8.9%)	81,823 (91.1%)	89,811 (100%)	
Age (mean)	82.8	83.7	83.7	(−1.06, −0.77)
Sex (Women %)	64.8%	66.9%	66.7%	(−0.03, −0.01)
Prevalence of assistive product use	83.7%	88.6%	88.2%	
Self-rated health status (Quite or very poor)	27.3%	23.3%	23.6%	(0.03, 0.05)
Living alone	63.0%	76.9%	75.6%	(−0.15, −0.13)
Proxy responses	65.4%	39.6%	42.0%	(0.25, 0.27)
Use of assistive product for				
seeing and reading	2.6%	5.0%	4.8%	(−0.03, −0.02)
hearing	18.3%	20.5%	20.0%	(−0.03, −0.01)
communicating	1.1%	1.1%	1.1%	(−0.002, 0.003)
mobility	68.4%	80.0%	79.0%	(−0.13, −0.12)
hygiene	61.8%	65.8%	65.5%	(−0.05, −0.03)
remembering	22.7%	7.3%	8.7%	(0.14, 0.16)
Other prescribed assistive product	4.9%	4.9%	4.9%	(−0.01, 0.01)
Safety alarm	61.9%	67.3%	66.9%	(−0.07, −0.04)

* 95% confidence interval (CI) of the difference in proportion and mean (for Age) between people with and without dementia.

**Table 3:** Estimated odds-ratios (95% CI in the parentheses) from binary logistic regression ^1^.

Outcomes	Covariates				
	Age For Each Year Above 65	Sex Women vs. Men	Health Poor vs. Good	Dementia With vs. Without	Living Alone vs. With Someone
Use of assistive product for					
seeing and reading	1.05 *** (1.05, 1.06)	1.27 *** (1.18, 1.37)	1.23 *** (1.14, 1.33)	0.56 *** (0.48, 0.65)	1.16 *** (1.06, 1.27)
hearing	1.09 *** (1.09, 1.09)	0.90 *** (0.86, 0.93)	1.08 *** (1.04, 1.13)	0.96 (0.90, 1.02)	1.02 (0.98, 1.07)
communicating	1.04 *** (1.03, 1.05)	0.77 *** (0.67, 0.89)	1.73 *** (1.49, 2.00)	1.02 (0.80, 1.29)	1.02 (0.86, 1.20)
mobility	1.03 *** (1.03, 1.04)	1.45 *** (1.40, 1.50)	2.05 *** (1.96, 2.15)	0.53 *** (0.50, 0.56)	0.94 ** (0.90, 0.98)
hygiene	1.02 *** (1.02, 1.02)	1.42 *** (1.38, 1.47)	1.86 *** (1.79, 1.93)	0.81 *** (0.77, 0.85)	0.84 *** (0.81, 0.87)
remembering	1.03 *** (1.03, 1.04)	1.12 *** (1.06, 1.19)	1.20 *** (1.13, 1.27)	4.30 *** (4.04, 4.58)	2.05 *** (1.91, 2.21)
Other prescribed assistive product	0.99 *** (0.98, 0.99)	0.98 (0.91, 1.05)	1.34 *** (1.24, 1.43)	0.98 (0.88, 1.10)	0.96 (0.89, 1.03)
At least one assistive product	1.04 *** (1.04, 1.04)	1.51 *** (1.44, 1.58)	2.30 *** (2.16, 2.45)	0.65 *** (0.60, 0.69)	0.92 *** (0.87, 0.96)
Participation in selecting assistive product(s) ^2^	0.99 * (0.99, 1.00)	1.17 *** (1.10, 1.25)	0.89 *** (0.83, 0.95)	0.80 *** (0.73, 0.88)	0.80 *** (0.75, 0.86)
Safety alarm	1.03 *** (1.03, 1.04)	1.17 *** (1.13, 1.20)	1.11 *** (1.07, 1.15)	0.84 *** (0.80, 0.88)	1.37 *** (1.32, 1.41)

* *p* ≤ 0.05, ** *p* ≤ 0.01, *** *p* ≤ 0.001. ^1^ The odds ratios are estimated by fitting a separate logistic regression model for each response variable (shown along the rows). ^2^ The response options were dichotomized; Yes was recoded as 1, and No and Partly were recoded as 2.

**Table 4:** Estimated cumulative odds ratios from cumulative logit models (95% confidence interval in parenthesis).

	Cumulative Odds Ratio (COR, In Inverse Scale *) From the Model For					
Covariate	Anxiety Yes vs. No	Safety Unsafe vs. Safe	Loneliness No vs. Yes	Respect No vs. Yes	Treatment No vs. Yes	Facilitation No vs. Yes
Dementia (Ref. no dementia)	1.28 (1.21, 1.34)	1.14 (1.08, 1.20)	0.73 (0.69, 0.76)	1.14 (1.08, 1.20)	1.06 ^ns^ (0.996, 1.12)	1.24 (1.14, 1.34)
Sex (Woman) (Ref. Man)	1.46 (1.41, 1.51)	1.07 (1.04, 1.11)	0.85 (0.83, 0.88)	0.99 ^ns^ (0.96, 1.02)	1.04 ^ns^ (0.999, 1.08)	0.87 (0.83, 0.92)
Age (Years −65)	0.98 (0.98, 0.98)	1.01 (1.01, 1.01)	1.00 (0.996, 1.00)	1.00 (0.99, 0.998)	0.98 (0.98, 0.98)	0.99 (0.99, 0.99)
Proxy response (Ref. self-response)	1.50 (1.45, 1.55)	1.40 (1.35, 1.45)	0.27 (0.26, 0.28)	1.44 (1.39, 1.48)	1.57 (1.51, 1.64)	1.01 ^ns^ (0.96, 1.06)
Living alone (Ref. Living not alone)	1.08 (1.04, 1.12)	2.69 (2.60, 2.79)	0.40 (0.39, 0.42)	1.41 (1.36, 1.46)	2.07 (2.00, 2.15)	1.01 ^ns^ (0.95, 1.07)
Poor health (Ref. Good or OK)	4.13 (3.98, 4.28)	1.09 (1.03, 1.14)	0.85 (0.81, 0.90)	1.94 (1.88, 2.01)	1.42 (1.34, 1.51)	1.87 (1.78, 1.97)
Use of assistive product(s) (Ref. No use)	1.08 (1.02, 1.13)	1.16 (1.12, 1.19)	0.61 (0.59, 0.63)	1.22 (1.16, 1.28)	1.51 (1.45, 1.56)	-
Safety alarm (Ref. No alarm)	1.02 ^ns^ (0.99, 1.05)	0.96 (0.93, 0.99)	-	-	-	-
Participation (Ref. Yes): Partly	-	-	-	-	-	2.78 (2.64, 2.93)
No	-	-	-	-	-	3.05 (2.82, 3.30)
Deviance (N)	116,100.9 (68,971)	141,591.1 (65,095)	126,426.8 (67,342)	143,432.1 (67,471)	92,215.1 (70,101)	46,491.1 (54,754)

* COR’s are reported in inverse scale in accordance with [20]. A value of COR greater than one indicates higher odds towards higher response category. For example, for Anxiety, “Yes” includes “Yes, slightly” and “Yes, severely”, and “No” includes “No.” Response categories were ordered according to their numeric coding scheme (see Table 1). ^ns^ Not significant at 5% level. The 95% confidence intervals are computed on basis of normal approximation, which is reasonable for large sample size.

## Data Availability

For information about the NBHW registers on patients and prescribed drugs, see https://www.socialstyrelsen.se/en/statistics-and-data/registers/ accessed 25 August 2022. For information about the NBHW register on care and services for older people and persons with impairments, see https://www.socialstyrelsen.se/statistik-och-data/register/aldre-och-personer-med-funktionsnedsattning/ (in Swedish) accessed 25 August 2022.
